# Mn Pretreatment Improves the Physiological Resistance and Root Exudation of *Celosia argentea* Linn. to Cadmium Stress

**DOI:** 10.3390/ijerph20021065

**Published:** 2023-01-06

**Authors:** Shaohong You, Zhenliang Deng, Mouyixing Chen, Yingyi Zheng, Jiu Liu, Pingping Jiang

**Affiliations:** 1 College of Environmental Science and Engineering, Guilin University of Technology, Guilin 541004, China; 2 Guangxi Key Laboratory of Environmental Pollution Control Theory and Technology, Guilin University of Technology, Guilin 541004, China; 3 College of Earth Sciences, Guilin University of Technology, Guilin 541004, China; 4 Guangxi Key Laboratory of Exploration for Hidden Metallic Ore Deposits, Guilin University of Technology, Guilin 541004, China

**Keywords:** Mn pretreatment, cadmium, *Celosia argentea* Linn., citric acid, root exudates

## Abstract

Phytoextraction using *Celosia argentea* Linn. by Mn pretreatment can potentially decontaminate Cd-contaminated soils. However, the mechanism that accelerates the Cd bioaccumulation is still unknown. In order to study the effect and mechanism of Mn pretreatment on Cd bioaccumulation in *C. argentea*, the hydroponic experiments were set to determine the chlorophyll content, antioxidant enzyme activity, malondialdehyde content, and root exudation of *C. argentea*. The results indicated that after seven days of Mn pretreatment, both the biomass and Cd concentrations in plants increased compared to the control group. One of the mechanisms for this was the improvement in the physiological resistance of *C. argentea* following pretreatment with Mn. Compared with Cd stress alone, Mn pretreatment increased photosynthesis and reduced membrane lipid peroxidation. Meanwhile, the activities of superoxide dismutase (SOD), peroxidase (POD), catalase (CAT), and ascorbate peroxidase (APX) were significantly reduced in leaves of *C. argentea* after Mn pretreatment through the reduction in the production of reactive oxygen species. In addition, Mn promoted the exudation of organic acids in the roots of *C. argentea*. The contents of citric and malic acids increased by 55.3% and 26.4%, respectively, which may be another important reason for Mn pretreatment increasing Cd bioaccumulation in C. argentea. Therefore, the present work shows that the pretreatment of seedlings with Mn can provide a meaningful strategy to improve the remediation efficiency of Cd-contaminated soils by *C. argentea*.

## 1. Introduction

Cadmium (Cd) is a hazardous heavy metal because of its high toxicity to humans and plants, even at low concentrations. In plants, Cd toxicity retards the growth rate and photosynthesis and increases oxidative damage and levels of reactive oxygen species (ROS) [1,2]. In humans, it causes multiple health complications, such as renal tubular dysfunction, heart disease, vascular problems, and cancer [3]. The well-known “*itai-itai*” disease is caused by Cd poisoning from Cd-contaminated rice [4]. Therefore, it is necessary to find an effective method to remediate Cd-contaminated soil.

Phytoremediation is regarded as an effective method for the treatment of heavy metal pollution of soils because it is environmentally friendly, allows in situ remediation, and is cost-effective. This technology involves the use of hyperaccumulators to remove heavy metals from soils. So far, 721 types of plants have been identified as hyperaccumulators [5], but only *Phytolacca americana* Linn. and *Celosia argentea* Linn. have been identified as Cd/Mn co-hyperaccumulators [6]. The effects of the interaction of Mn and Cd have been widely studied in plants. Earlier studies suggested that Mn exhibits an antagonistic role and reduces Cd bioaccumulation as well as its associated toxicity in rice [7]) and in *Phytolacca acinose* Roxb [8]. Liu et al. [9] found that the addition of Mn decreased the bioaccumulation of Cd in *C. argentea* grown in hydroponic experiments, whereas the opposite findings were recorded from the soil systems. The main reason for this contradiction is that Cd and Mn compete for the same transporters in solution systems while they compete for soil adsorption sites in soil systems. All of the above studies were conducted under environmental conditions of the coexistence of Mn and Cd. However, only a few studies have been carried out on the effect of Cd bioaccumulation following pretreatment with Mn. Fu [10] reported that Mn pretreatment of rice improved the Cd uptake in both roots and stems. Results from Jiang et al. [11] also showed that, following Mn pretreatment, the net Cd2+ flux to the roots of *C. argentea* was also increased compared with the group without Mn pretreatment. These results indicate that plants pretreated with Mn may increase Cd uptake and so could enhance the efficiency of soil Cd phytoremediation by *C. argentea*. The efficiency of hyperaccumulators was the limiting factor for its implication in heavy metal-contaminated soils. There were numerous ways proven to a useful way to improve the phytoremediation rate, including the addition of exogenous organic acids [12], plant growth-promoting rhizobacteria [13], *Bacillus megaterium* [4], *Pseudomonas aeruginosa* [14]. Nowadays, we found that the plants pretreated by Mn could be a new method to enhance the phytoremediation rate of Cd-contaminated soil. However, the mechanism that accelerates this bioaccumulation process has been largely unexamined and is worth further investigation and discussion.

Relevant studies have shown that plants treated with moderate Mn, auxin (IAA), gibberellin 3 (GA3), and zeatin (ZT) in *C. argentea* increased significantly, which promoted the growth of *C. argentea* [15]. Therefore, Mn alters the plant hormone contents and increased plant growth, which increases the Cd accumulation. Mn is an essential trace element for optimum growth and development and acts by regulating various physiological processes in plants, such as chlorophyll (Chl) synthesis and regulation, as well as lipid peroxidation [1,16]. For example, Cd stimulates malondialdehyde (MDA) content, which governs lipid peroxidation [17], whereas Mn supplementation can reduce MDA accumulation in plants [8]. In addition, it has been shown that Cd inhibits photosynthetic permanence and reduces the content of both Chl a and Chl b [18], whereas Mn supplementation relieves these inhibitory effects [1]. However, whether Mn pretreatment lessens Cd toxicity by increasing physiological endurance, thus reducing Cd toxicity and increasing Cd uptake in plants, is unknown. Moreover, root exudates of low molecular weight organic acids (LMWOAs), such as citric, malic, oxalic, and succinic acids, have been identified as key regulators of plant Cd uptake and accumulation [12,19]. Specifically, these organic acids can react with numerous metal ions in both hydroponic and soil systems, increasing the mobility of the metals in the rhizosphere region and, therefore, enhancing the Phyto-availability of heavy metals to plants [20]. Yu et al. [12] reported that supplementation with LMWOAs, such as tartaric acid, malic acid, and citric acid, significantly enhanced Cd uptake and subsequent bioaccumulation by *C. argentea*. As a result, plants pretreated with Mn show considerably increased root exudation of organic acids and boosted the bioaccumulation of Cd.

Based on these studies, we speculated that following Mn pretreatment plants exhibit elevated photosynthetic performance, reduced lipid peroxidation, as well as improved root exudation of LMWOAs, and thus increase Cd bioaccumulation in *C. argentea*. Therefore, the primary objectives of this work were to further investigate this hypothesis by examining the following: (1) the role of Mn pretreatment in improving the physiological resistance to *C. argentea*; and (2) the role of Mn pretreatment in increasing the exudation of organic acids in roots of *C. argentea*.

## 2. Materials and Methods

### 2.1. Seedling Culture

Seeds of *C. argentea* were collected from the heavy metal remediation research center (Yangshuo County, Guilin, Guangxi) and were subsequently placed into 10% hydrogen peroxide (H_2_O_2_) solution for 10 min after soaking in ultrapure water overnight. The seeds were rinsed with deionized water and spread on seedling trays filled with nutrient soil. Afterward, seedling cultures were placed in a greenhouse with 60% relative humidity at a day/night temperature range of 25/18 °C. Following the development of approximately 4–6 true leaves and an average height of 5–6 cm, similarly sized seedlings were transplanted to the experimental plots to conduct hydroponic experiments.

### 2.2. Hydroponic Experiments

#### 2.2.1. Hydroponic Experiment 1

Seedlings were transplanted into 1 L plastic pots containing 1/2 Hoagland solution. Then, Mn (supplied as MnSO_4_·H_2_O) was added into the solution to give a concentration of 50 µM at treatment times of 0 (control group), 1, 3, 5, and 7 days, respectively. Later, the seedlings were transferred into 5 µM Cd solution (without Mn), which was supplied as CdCl_2_·2.5H_2_O. The plants were harvested after one month of culture and subsequently used to determine the dry weight, Cd concentration, Chl content, antioxidant enzyme content, and MDA content. Each treatment was performed in triplicate, and the solution medium was changed every 5 days.

#### 2.2.2. Hydroponic Experiment 2

To study the effect of Mn pretreatment on the root exudation of organic acids, the seedlings were cultured under hydroponic conditions with 0 (control group) and 50 µM Mn, respectively. The plants were harvested after 7 days of culture, and each treatment was performed in three duplicates.

### 2.3. Chemical Analyses

First, the plant content was washed with deionized water and separated into roots, stems, and leaves, and then dried at 70 °C to a constant weight to obtain the dry weight. Then, 0.2 g of the sample was weighed and digested with HNO_3_ + H_2_O_2,_ following dilution with deionized water to 50 mL. The Cd concentration was determined using inductively coupled plasma-mass spectrometry (ICP-MS) (Perkin-Elmer NexION, 2000B, USA).

The activities of superoxide dismutase (SOD), peroxidase (POD), catalase (CAT), and ascorbate peroxidase (APX) were measured as described by Jiang et al. [21]. The MDA content in roots and leaves was measured with a modified thiobarbituric acid–malondialdehyde assay [22]. To extract Chl from leaves (about 0.2 g) in *C. argentea*, a 1:1 mixture of absolute ethanol and acetone was used. The Chl content was determined by measuring the absorbance values at 645 and 663 nm, respectively [22].

The collection and analysis methods for root exudates were performed according to a previously published protocol [23]. The roots were washed three times with ultrapure water and immersed in 500 mL ultrapure water for 2 h in the growth chamber to collect root exudate samples. After collection, the root exudates were stored at −20 °C prior to analysis. The quantification of LMWOAs was performed using a liquid chromatography-mass spectrometry (LC-MS) XSelect HSS T3 column (250 mm x 4.6 mm, 5 μm) with 40 Mm KH_2_PO_4_–H_3_PO_4_ (pH = 2.40) as the mobile phase, at a flow rate of 1.0 mL/min, and 25 °C and 205 nm wavelength.

### 2.4. Statistical Analysis

The mean and standard errors for the data were analyzed using SPSS 18.0 software. The data were subjected to one-way analysis of variance (ANOVA) using SPSS 18.0 for statistical significance at *p* < 0.05 and *p* < 0.01. The figures and tables were produced using OriginPro 2020 and Microsoft Excel 2010.

## 3. Results and Analysis

### 3.1. Mn Pretreatment Increased the Biomass of C. argentea

**Figure 1:**
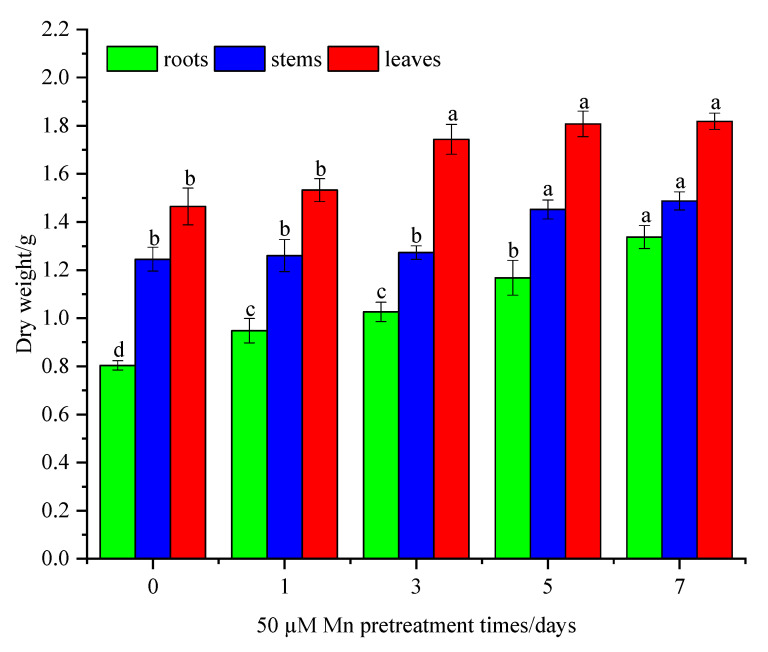
Biomass of *C. argentea* under Cd stress after exposure to different times of Mn pretreatment. Results are presented as mean values ± SD (standard deviation) (*n* = 3). Different letters on the error bars indicate statistically significant differences according to the least significant difference (LSD) test (*p* < 0.05).

After different Mn pretreatment days, the biomass of plants was determined after one month of Cd culturing. The results showed that Mn pretreatment of *C. argentea* increased the biomass of the roots, stems, and leaves compared to the control group (Figure 1). The biomass of the leaves increased by 19.2%, 24.0%, and 24.7% after Mn pretreatment on three, five, and seven days, respectively, relative to the control group. Similarly, the biomass of stems increased by 16.0% and 18.4% at five days and seven days with Mn pretreatment versus the group without Mn pretreatment. Significant differences were identified in the root biomass in the Mn-pretreated groups compared to the control groups, with respective increases of 17.5%, 27.5%, 45.0%, and 66% after one, three, five, and seven days.

### 3.2. Mn Pretreatment Stimulated Cd Accumulation of C. argentea

**Figure 2:**
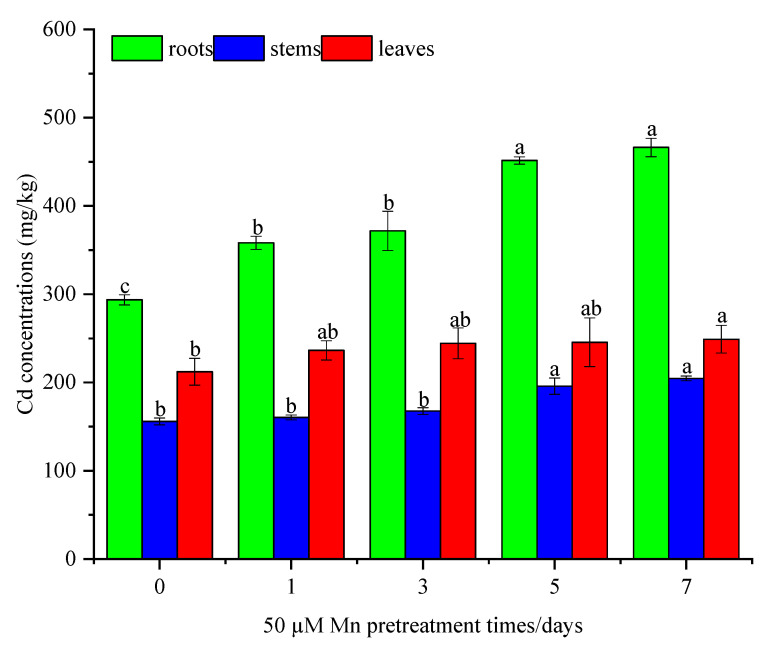
Cd concentrations in roots, stems, and leaves of *C. argentea* under Cd stress at different Mn pretreatment times. Results are presented as mean values ± SD (*n* = 3). Different letters on the error bars indicate differences that are statistically significant according to the LSD test (*p* < 0.05).

The Cd concentrations in roots, stems, and leaves were determined after acquiring the biomass. Compared with the Cd stress only, the Cd concentration in roots and leaves increased significantly after different Mn pretreatment times (Figure 2). The Cd concentrations in stems increased significantly after five days of pretreatment with Mn. All Cd concentrations reached their largest values in roots, stems, and leaves after seven days of Mn pretreatment, with the concentrations being 466.25 ± 10.40 mg/kg, 204.63 ± 2.65 mg/kg, and 249.00 ± 15.64 mg/kg, respectively. Compared to the control group, the Cd concentration in roots, stems, and leaves increased by 58.7%, 31.2%, and 17.3%, respectively.

### 3.3. The Effects of Mn Pretreatment on the Chlorophyll Content of C. argentea under Cd Stress

**Figure 3:**
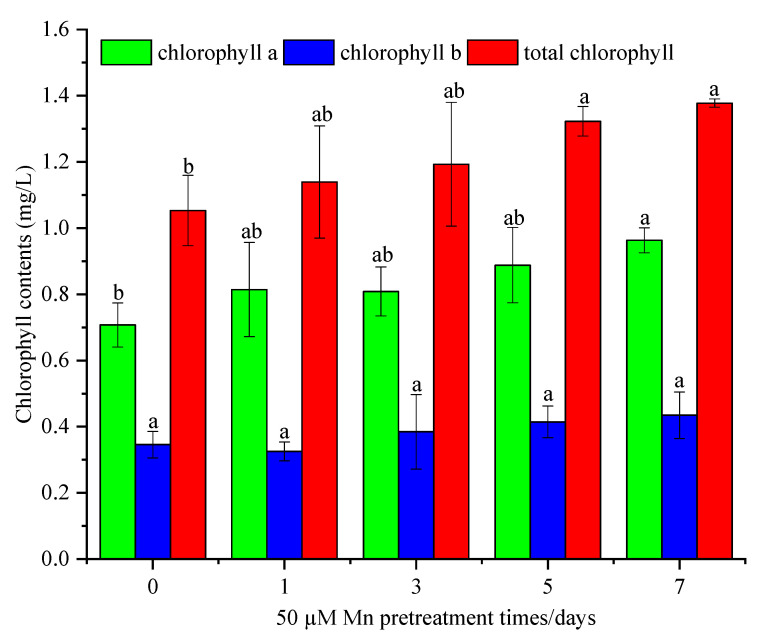
The Chl contents in leaves of *C. argentea* under Cd stress after different Mn pretreatment times. The results are presented as mean values ± SD (*n* = 3). Different letters on the error bars indicate statistically significant differences according to the LSD test (*p* < 0.05).

Following the different Mn pretreatment durations and a month of Cd culture, the contents of Chl a, Chl b, and total Chl in the leaves of *C. argentea* were determined. As shown in Figure 3, the contents of Chl a, Chl b, and total Chl increased following Mn pretreatment at different time intervals. As expected, the total Chl contents increased by 7.6%, 13.3%, 25.7%, and 30.5% after one, three, five, and seven days of Mn pretreatment, respectively, compared to the control group. Consistent with the trend in total Chl, a similar trend was observed for Chl a and Chl b. However, no difference was found in Chl b levels between the control group and the Mn pretreatment group.

### 3.4. Mn Pretreatment Caused a Drop in Antioxidant Enzyme Performance and MDA Content in C. argentea under Cd Stress

**Figure 4:**
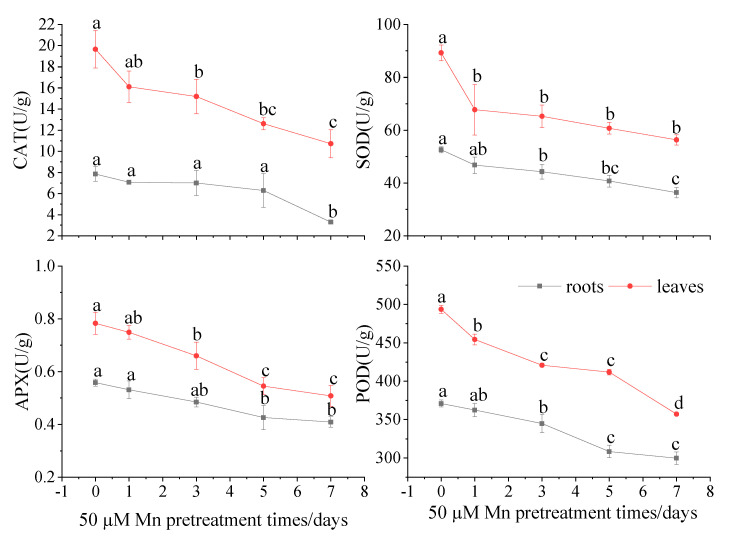
The effects of different Mn pretreatment times on antioxidant enzyme activities in roots and leaves of *C. argentea* under Cd stress. Results are presented as mean values ± SD (*n* = 3). Different letters on the error bars indicate statistically significant differences according to the LSD test (*p* < 0.05).

After the different Mn pretreatment durations and Cd culture, the antioxidant enzyme activities in the roots and leaves were determined. The results showed that the activities of CAT, SOD, APX, and POD decreased with increasing treatment times (Figure 4). The leaves of *C. argentea* were distinguished by elevated antioxidant enzyme performance compared with the root tissues. Compared to the control group, the CAT activities in the leaves decreased by 18.1%, 22.7%, 35.9%, and 45.5% after 1, 3, 5, and 7 days of Mn pretreatment, respectively. However, the CAT activity had fallen by 58.1% in roots after seven days of Mn pretreatment. The SOD activities in leaves decreased by 24.1%, 26.9%, 32.1%, and 36.9%, that in roots decreased by 11.2%, 15.3%, 22.6%, and 30.9%, after 1, 3, 5, and 7 days of Mn pretreatment, respectively, compared to the control group. In addition, APX activities in the leaves and roots were significantly reduced after 5 and 7 days of Mn pretreatment (23.6%, 26.7%) versus the control group (30.3%, 35.1%), respectively. The POD activities in the leaves and roots decreased by 27.7% and 19.2%, respectively, after 7 days of Mn pretreatment compared to the control group.

**Figure 5:**
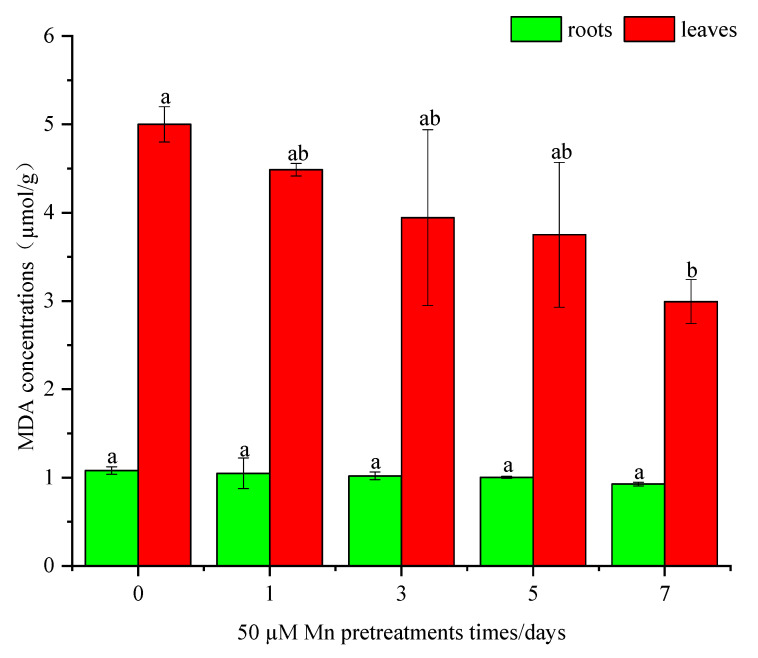
The effects of different Mn pretreatment supplementation times on MDA contents in roots and leaves of *C. argentea* under Cd stress. Results are presented as mean values ± SD (*n* = 3). Different letters below on the error bars indicate statistically significant differences according to the LSD test (*p* < 0.05).

The MDA contents in the leaves showed a declining trend with increasing Mn pretreatment times (Figure 5). The MDA content had the highest value (5.00 µmol/g) in the control group, with a corresponding value of 2.99 µmol/g after seven days of Mn pretreatment. However, the MDA content in roots showed no significant difference between the control group and the Mn pretreatment group.

### 3.5. Mn Pretreatment Increased Organic Acids in the Root Exudates of C. argentea under Cd Stress

The root exudation of the plants was collected and determined after seven days cultured. The results showed that Mn pretreatment increased the secretion of organic acids in root exudates of *C. argentea* compared with the control group (Table 1). Four types of organic acid, including oxalic acid, malic acid, lactic acid, and citric acid, were present in the control group. However, seven different classes of organic acids were detected in roots treated with Mn. In addition, the malic acid and citric acid contents increased by 26.4% and 55.3%, respectively, in samples receiving Mn dosing, compared with the control group.

**Table 1:** The effects of Mn treatment on exudation of organic acids (μg/mL) in the root of *C. argentea*.

Organic Acids	Different Treatments	
	0 μM Mn (Control Group)	50 μM Mn
Oxalic acid	0.37 ± 0.12 b	0.49 ± 0.11 a
Tartaric acid	ND	ND
Malic acid	2.58 ± 0.67 b	3.26 ± 1.02 a
Lactic acid	1.25 ± 0.35 ab	1.74 ± 0.67 a
Acetic acid	ND	2.97 ± 0.99
Maleic acid	ND	0.01 ± 0.002
Citric acid	2.17 ± 0.88 b	3.37 ± 1.32 a
Fumaric acid	ND	0.01 ± 0.001

Results are means ± SDs (*n* = 3). Different letters on the error bars indicate statistically significant differences according to the LSD test (*p* < 0.05); ND, not detected.

## 4. Discussion

### 4.1. Mn Pretreatment Improves the Physiological Resistance of C. argentea, Thus Increasing Plant Biomass and Cd Bioaccumulation

Under Cd stress, the redox environment in plants is disrupted and produces a large number of ROS, which could cause membrane lipid peroxidation and destroy the normal metabolism of the plant. To regulate the level of ROS, plants produce antioxidative enzymes, including SOD, APX, CAT, and POD, to inhibit ROS production [21]. Similar trends were also presented in *C. argentea*, in which the antioxidative enzymes reached a maximum under Cd stress only (Figure 4). Mn pretreatment significantly reduced the prevalence of these enzymes. It means that more ROS were produced at Cd stress while the plants were pretreated by Mn, reducing the ROS production. Meanwhile, Cd could cause lipid peroxidation in plants and increase the MDA contents [17]. Similarly, the maximum MDA content was observed in the control group in this study. Therefore, Cd increased the membrane lipid peroxidation of *C. argentea*. Following, the MDA contents presented a decreasing trend with the different Mn pretreatments times. This implies that when pretreated with Mn, plants could decrease membrane lipid peroxidation, thereby reducing MDA content. In addition, the MDA content in leaves was higher than that in roots, which indicated that Mn pretreatment reduced peroxidation tolerance in leaves rather than in roots of *C. argentea*. This is also supported by the observations of Liu et al. [8], whereby Mn alleviated lipid peroxidation in leaves of *Phytolacca acinosa* by intracellular antagonism between Mn and Cd within the experimental plant units. Besides, Mn can reduce the toxicity of cadmium and thus protect the photosynthesis of plants from its effects. Zornoza et al. [24] reported that the bioaccumulation of Cd in white lupin (*Lupinus albus*) might be increased by moderate Mn treatment, which was shown to dramatically reduce Cd toxicity and improve photosynthesis. The Chl content reflects the capacity for photosynthesis of the plant. Some studies have reported that Cd inhibited photosynthesis in plants, while Mn accumulation in the chloroplasts of leaves of Cd-treated lettuce could reduce Cd toxicity with no visual effects [25,26]. In the present study, the total Chl content in leaves increased by 30.5% after seven days of Mn pretreatment compared to Cd stress only (Figure 3). These results indicated that Mn could reduce the Cd toxicity towards photosynthesis in plants. In total, the ROS and MDA content were two major factors that influenced the growth of plants caused by Cd toxicity. The photosynthesis of plants is the most important process of physiological, which may inhibit by Cd stress. Mn could alleviate these negative effects on plant growth caused by Cd. Therefore, Mn pretreatment increased plant biomass and Cd bioaccumulation by improving the physiological resistance of *C. argentea* through increased photosynthesis, reduced ROS production, and reduced membrane lipid peroxidation.

### 4.2. Mn Promotes the Exudation of Organic Acids from Roots, Including the Matric and Citric Acids, Thereby Increasing Cd Bioaccumulation in Plants

In plants pretreated with Mn, the exudation of organic acids increased compared with the control group (Table 1). This has also been verified by Mora et al. [27], who found that Mn pretreatment noticeably enhanced the organic acid content of root exudation in perennial ryegrass (*Lolium perenne* L.). This corresponds to the potential for organic acids to modify the pH and redox potential of the rhizosphere by chelating, complexing, and depositing heavy metals, as well as by altering the activity of rhizospheric microbes [28,29]. Consequently, organic acids in root exudates could alter the chemical speciation of heavy metals after subsequent stimulation of bioavailability. Luo et al. [28] found that organic acids increased Cd uptake and its bioaccumulation in *Sedum alfredii*. Similarly, Tao et al. [19] reported that LMWOAs increased the plant biomass and Cd uptake by maize. In this study, Mn promoted the exudation of organic acids, in which the relative content of citric and malic acids increased by 55.3% and 26.4% compared with the control group (Table 1). These two kinds of organic acids have received growing attention in recent years due to reports that they are powerful organic acids for expediting the elimination of heavy metals [20,30,31]. For example, 85% of ^109^Cd was transported in the shoots of *Sedum alfredii* by application of citric acid in 24 h, compared with 75% uptake in the control group [20]. The Cd bioaccumulation in the sunflower plants (*Helianthus annuus* L. cv. *Avante*) increased by 51.17% with the addition of citric acid [32]. What is more, it should be noted that the heavy metals initially bound to organic acids were later transported into the cell wall and vacuoles, thereby decreasing the heavy metal toxicity towards plants and with resultant enhanced bioaccumulation [33]. This was observed by Li et al. [34], where Cd bound with oxalic acid and citric acid subsequently migrated to the shoot cell walls in *Arabis alpina*, thus favoring decreased Cd toxicity with accompanying elevated Cd bioaccumulation. As a result of changing the chemical form of the heavy metal, LMWOAs have a significant impact on the bioaccumulation of Cd in plants. Mn pretreatment increases the exudation of organic acids, which could increase Cd bioavailability and reduce Cd toxicity and thus enhance Cd bioaccumulation in *C. argentea.*

## 5. Conclusions

The results presented here show that Mn pretreatment increased the biomass and Cd bioaccumulation in *C. argentea*. In our study, compared with Cd treatment alone, the SOD, POD, APX, and CAT activities were reduced following Mn pretreatment, indicating that Mn pretreatment reduced the production of ROS. Meanwhile, Mn pretreatment resulted in decreased MDA content in plants but increased total Chl content. What is more, Mn pretreatment increased the diversity and contents of organic acids in the root exudates, which had a positive effect in increasing Cd availability and decreasing Cd toxicity. Therefore, Mn pretreatment increased the physiological resistance and promoted the exudation of organic acids, thus increasing the biomass and bioaccumulation of Cd. This finding provided a new implication for the improvement of Cd phytoextraction.

## Data Availability

The data that support the findings of this study are available from the corresponding author upon reasonable request.
